# Oxygen Transport Membranes for Efficient Glass Melting

**DOI:** 10.3390/membranes10120442

**Published:** 2020-12-19

**Authors:** Luca Mastropasqua, Francesca Drago, Paolo Chiesa, Antonio Giuffrida

**Affiliations:** 1 Advanced Power and Energy Program, University of California, Irvine, CA 92697, USA; 2 RSE—Ricerca sul Sistema Energetico S.p.A., 20134 Milano, Italy; francesca.drago@rse-web.it; 3 Politecnico di Milano—Dipartimento di Energia, 20156 Milano, Italy; paolo.chiesa@polimi.it (P.C.); antonio.giuffrida@polimi.it (A.G.)

**Keywords:** oxygen transport membrane, LSCF, perovskite, glass melting, oxy-fuel combustion

## Abstract

Glass manufacturing is an energy-intensive process in which oxy-fuel combustion can offer advantages over the traditional air-blown approach. Examples include the reduction of NO_x_ and particulate emissions, improved furnace operations and enhanced heat transfer. This paper presents a one-dimensional mathematical model solving mass, momentum and energy balances for a planar oxygen transport membrane module. The main modelling parameters describing the surface oxygen kinetics and the microstructure morphology of the support are calibrated on experimental data obtained for a 30 μm thick dense La_0.6_Sr_0.4_Co_0.2_Fe_0.8_O_3-δ_ (LSCF) membrane layer, supported on a 0.7 mm porous LSCF structure. The model is then used to design and evaluate the performance of an oxygen transport membrane module integrated in a glass melting furnace. Three different oxy-fuel glass furnaces based on oxygen transport membrane and vacuum swing adsorption systems are compared to a reference air-blown unit. The analysis shows that the most efficient membrane-based oxyfuel furnace cuts the energy demand by ~22% as compared to the benchmark air-blown case. A preliminary economic assessment shows that membranes can reduce the overall glass production costs compared to oxyfuel plants based on vacuum swing adsorption technology.

## 1. Introduction

The term glass generically refers to high-viscosity substances, which do not possess enough mobility during solidification. Thus, they do not have sufficient time to form a regular crystal lattice, resulting in an intermediate metastable stage marked by the formation of an amorphous solid, which lacks the geometrical order typical of crystal lattices [1]. Common raw materials for the glass production are silica (SiO_2_), sodium carbonate (Na_2_CO_3_), limestone (CaCO_3_) and, dolomite (CaMg(CO_3_)_2_). However, the actual composition varies significantly, depending on the glass type. These raw materials are heated in a furnace to high temperatures to form molten glass. The molten glass undergoes further processing to originate the final product [2]. 

A furnace operates glass melting at very high temperature (>1400–1500 °C) in a confined space surrounded by refractory material. The heat input is generally supplied by burning oil or natural gas in air. Electric booster systems can be installed to supply additional energy to increase the melting capacity. The conventional arrangement of a glass furnace includes a regenerative heat exchanger consisting of two refractory chambers. One chamber is used to store heat from the flue gas, at the same time the other chamber is used to preheat the combustion air. The paths of flue gas and combustion air through the regenerator chambers are interchanged every 15–20 min. The raw material is continuously introduced in the furnace through a feeding port, while molten glass is continuously removed from the furnace [3,4].

The theoretical energy requirement consists of the endothermic heat for the glass reaction, the sensible heat for glass heating and the sensible heat for batch gases (gases from glass reaction). According to the theoretical heat requirement reported by Sardeshpande et al. [5], the soda-lime glass (composed mainly of silicon dioxide (71–75%), sodium oxide (12–16%) and calcium oxide (10–15%)) needs 2671 kJ kg^−1^ of glass, if no cullet (i.e., crushed glass ready to be re-melted) is used. On the other hand, the theoretical minimum is 1886 kJ kg^−1^ if the furnace operates on 100% cullet. Such a reduction is due to the absence of the reaction heat of the virgin raw material (487 kJ kg^−1^) and the sensible heat of batch gases (298 kJ kg^−1^).

The use of cullet is beneficial since it is cheaper than virgin raw material and allows energy savings. Moreover, the substitution of raw materials by cullet reduces CO_2_ process emissions, which are released by the decomposition of carbonates in raw materials. In the industrial practice, external cullet is extensively used only in the container glass and glass wool production [2].

**Figure 1:**
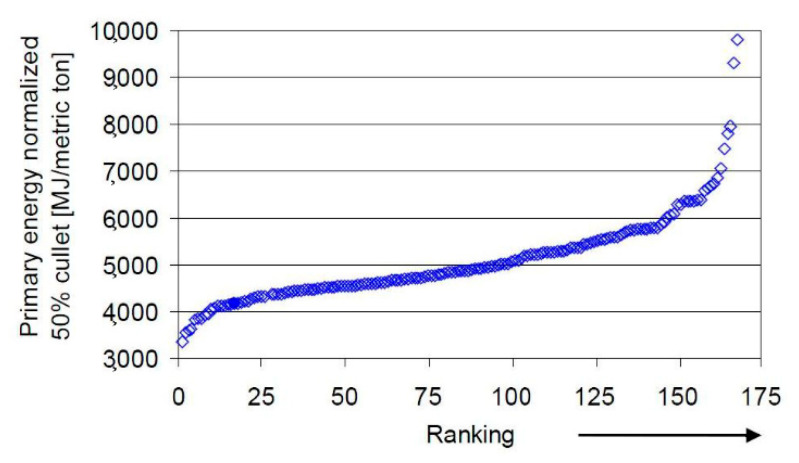
Example of ranking of energy efficiency from most efficient to lowest efficient container glass furnace of a set of 168 furnaces. Each point refers to one existing container glass furnace. Specific energy data are normalized to 50% cullet and the primary energy consumption takes electricity and oxygen production into account (adapted from Reference [6]).

The above mentioned theoretical heat requirements correspond to the lowest thermodynamic limits [5] but even well insulated glass furnaces will be affected by some structural heat losses. In addition, fossil fuel firing is always associated with flue gas production. Although the flue gas heat content can be partly recovered by combustion air and batch preheating, flue gas is released at temperature of at least 200–250 °C. Thus, the minimum achievable energy consumption level is unavoidably greater than the one reported by Sardeshpande et al. [5]. Energy benchmarking for glass furnaces has been thoroughly discussed by Beerkens [6,7,8]. Referring to the most recent paper [6], Figure 1 shows the ranking of the energy efficiency of 168 container glass furnaces, starting from the lowest specific energy consumption (most energy efficient) up to the highest specific energy consumption. The primary energy values are normalized for the case of 50% recycling cullet in the batch and account for the contribution energy required for electric power generation in case of electric boosting and oxygen production. According to the data in Figure 1, the minimum achievable energy consumption level for normal batch would be around 3.8 MJ kg^−1^, which reduces to around 2.5 MJ kg^−1^ for 100% cullet.

Oxygen-firing is an effective solution for saving energy in glass furnaces. In the Beerkens’ benchmark study [8], the average specific energy consumption (4.3 MJ kg^−1^) of oxygen-fired container glass furnaces is lower than that of new end-port fired regenerative furnaces (4.6 MJ kg^−1^ including the energy demand for oxygen production) using 50% cullet. Oxygen-fired furnaces with cullet and/or batch preheating also showed values in the range 3.4–3.6 MJ kg^−1^.

Different solutions can be implemented to supply oxygen to a glass furnace. In the conventional scenario, oxygen is delivered by a pressure swing adsorption (PSA)/vacuum swing adsorption (VSA) unit (which represents the most convenient technology in relation to oxygen demand and purity) or cryogenic distillation processes. An emerging alternative for oxygen separation is represented by membranes based on mixed ionic and electronic conducting (MIEC) materials that promise to reduce the energy consumption of oxygen separation compared to conventional technologies. Since they operate at temperature around 1000 °C, an effective thermal integration in high-temperature processes is a key aspect to achieve an efficient oxygen separation. Glass melting, that takes advantage from oxygen combustion and it is operated at temperature significantly above 1000 °C, is one of the most interesting industry for application of these membranes. This paper investigates this topic by an extended approach ranging from implementation and calibration of a newly developed model for performance evaluation of membrane modules, to heat and mass balance simulation of glass melting furnaces based on alternative technologies, to a comparative economic assessment. 

## 2. Review of Oxygen Transport Membranes

The main purpose of Oxygen Transport Membranes (OTM) is to separate O_2_ throughout a non-galvanic electrochemical mechanism occurring within an O_2_-ion transport dense membrane, that is, without the application of an external voltage and the creation of an electric current in an external circuit. The driving force of the process is solely dependent on the difference of chemical potential of O_2_ between the two sides of the membrane (i.e., O_2_ partial pressure driven OTM) [9]. Sunarso et al. 2008 [10] reviews dense ceramic membranes for O_2_ transport. The study identifies the main characteristics of fast oxygen-ion conducting material: (i) fast oxygen-ions exchange at the relevant gas/solid interfaces (i.e., high ionic conductivity in the temperature range of interest for the final application); (ii) high catalytic activity for the Oxygen Reduction Reaction (ORR); (iii) adequate microstructure morphology (i.e., particle and pore size, porosity and tortuosity) to promote O_2_-ions diffusion throughout the material pores; (iv) chemical and physical stability in oxidizing and reducing atmosphere during fabrication and operation. Contrary to galvanic systems in which an external electrical circuit is present, OTMs must ensure a good electrons transport within the dense active material to respect the overall charge neutrality criteria (i.e., simultaneous flux of all charge carriers - ions and electrons - involved in the electrochemical reactions) [11].

In Mixed Ionic-Electronic Conductor (MIEC)-based membranes, charge transport processes are characterized by an increased mobility of electron holes ($h^{•}$), other than only ion vacancies ($V_{O}^{••}$), that is, ionic transference number $t_{i}<1$. Therefore, MIEC materials are characterized by both high oxide-ion and electron conductivities. This can either be achieved by employing single-phase materials with mixed conducting properties, such as the oxygen-deficient perovskite structure La_1−x_Sr_x_Co_1−y_Fe_y_O_3-δ_ (LSCF) [12] or by dual-phase membranes, which combine—in a percolated network—materials that show good ionic and electronic conduction properties [13]. In the former case, charged particle transport occurs in the dense membrane bulk without the necessity of a dense Triple Phase Boundary (TPB), since the active sites are distributed across the thickness of the membrane. Consequently, it is possible to speak of Double Phase Boundary (DPB)—i.e., point of contact between the active dense phase and the gas phase—when dealing with MIEC materials [14]. In case a MIEC material is employed, the following requirements should be verified simultaneously: (i) high O_2_-ion conductivity to supply the membrane surface with oxygen vacancies as soon as they are occupied by fresh new $O_{0}^{X}$; (ii) high electronic conductivity to ensure that electron holes diffusion does not become a rate limiting step in the ion transport process, as also reported by Chen et al., 1995 [15].

From a manufacturing perspective, it is crucial to be able to lay extremely thin dense membrane layers (i.e., 10–50 µm-scale) as this would increase the permeation performance of the membrane proportionally to the decrease in its thickness [16]. This requirement contrasts with the thermo-mechanical resistance capabilities of the membrane when this is subjected to pressure and temperature gradients. Typical industrial applications—such as oxy-combustion [17,18], carbon capture [19] and O_2_ separation [20]—require the operation at high pressure and temperature; therefore, membrane’s degradation and thermo-mechanical resistance represent important features for a successful process integration. For this reason, ceramic or metallic porous support structures are usually employed to operate as structural resistance, possibly without impeding or hindering the diffusive transport of gaseous species [21].

This work aims to study the integration of OTM for production of pure oxygen and enabling oxy-fuel combustion into the glass industry. Herein we propose to study the thermodynamic performance and economic viability of glass melting furnaces with oxy-fuel combustion via OTM to achieve lower specific energy consumption and higher energy density compared to conventional glass melting furnaces. The first section of this study focuses on the developing of a new 1-D finite volume model of a co-flow and counter-flow planar oxygen transport membrane. The model is then used to design complete OTM modules for oxygen separation and integration into different glass furnace configurations. The second half of this work describes the system thermodynamic analysis and economic assessment of OTM-based glass furnaces. The model is in-house developed at Politecnico di Milano in a Fortran 90 environment. A previous version of the model has already been employed for designing reactive OTMs for small scale H_2_ production from natural gas [22]. The model is calibrated against experimental data obtained from the FP7 European Project GREEN-CC using a 30 μm thick dense LSCF membrane layer, supported on a 0.7 mm porous LSCF structure. The model solves the mass, momentum, and energy balances in the membrane’s channels, which are subdivided into finite control volumes by a computational grid. The main outcome of the model is the amount of permeated oxygen given the user-defined geometrical (e.g., number of channels, membrane layer and support thicknesses) and thermochemical (e.g., temperature, pressure, composition) inputs in the membrane reactor.

The novelties of this study are:

- A new 1-D OTM model is developed to predict the thermodynamic performance and design of planar membrane modules;
- The OTM model is calibrated using newly published experimental data from a 30 μm thick dense LSCF membrane;
- The designed OTM modules are integrated into three new glass furnace layouts with oxy-fuel combustion;
- The proposed layouts are assessed from an energy and economic viewpoint to select the optimal configuration.

## 3. Modelling of Supported OTM

The membranes considered in this work are characterized by planar channels with rectangular cross-sectional area. A single membrane channel couple (i.e., feed and sweep sides) is considered in the simulations. Each channel is discretized into control volumes by a uniformly spaced computational grid.

### 3.1. OTM Module Model

The permeation model, the fluid-dynamic model and the heat transfer model are included into a one-dimensional (1D) OTM module model, which aims at designing a complete membrane stack. The OTM model calculates the total surface area required to supply a given oxygen demand and then subdivides the area into layers, stacks, and modules. Various parallel channels constitute each membrane layer, which then form a stack unit. Additionally, depending on the type of application and its design requirements (e.g., the acceptable pressure losses), the integration in series or in parallel of different stacks makes an OTM module.

The development of the OTM model is based on the following assumptions:

- One-dimensional analysis: numerical discretization in finite volumes along the axial directory of the streams. In each finite volume, the permeation model, the fluid-dynamic models are solved
- Steady state analysis
- Ideal gases and ideal mixtures
- Uniform distribution across all channels
- Negligible thermal losses towards the environment

The OTM module model is then used to design membrane modules for glass furnaces and evaluate their performance in various operating conditions—i.e., with and without sweep gas, atmospheric and pressurized operation.

### 3.2. Fluid-Dynamic and Heat Transfer Model

The fluid-dynamics model estimates the viscous friction, which determines the pressure losses within the channels in the feed and permeate compartments. The model accounts for both laminar and completely developed turbulent flow regimes; the former regime is modelled using an interpolation function of tabulated data taken from Reference [23], valid for different channel cross sectional shapes and height/width ratios. The latter is modelled with the Petukhov’s correlation:

(1) $$f={\left[0.790\cdot ln\left(Re_{D}-1.64\right)\right]}^{-2}\;\{\begin{array}{c} 0.5\leq Pr\leq 2000\\ 3000\leq Re_{D}\leq 10^{6} \end{array}$$

The pressure losses for viscous effects are evaluated via the Darcy–Weisbach equation:

(2) $$\Delta p=\frac{1}{2}\cdot \rho \cdot {\bar{u}}^{2}\cdot f\cdot \frac{L}{D_{h}},$$

where, L is the total length of the considered finite volume, whilst $\bar{u}$ and ρ are considered at the volume inlet, assuming them constant within each node.

Finally, the steady state one-dimensional mass, momentum and energy balances are evaluated per each control volume for both feed and permeate sides.

(3) $$\frac{\partial \left(C_{i}v_{i}\right)}{\partial x}=\underset{j}{\sum }\nu_{j}r_{j}$$

(4) $$\frac{\partial \left(C_{i}v_{i}^{2}\right)}{\partial x}=-\frac{\partial p}{\partial x}$$

(5) $$\frac{\partial \left(C_{i}v_{i}h_{i}\right)}{\partial x}=\sum_{j}\Delta H_{r}{}_{j}^{{}^{\circ}}r_{j},$$

where, i represents either the feed, the permeate, the membrane or the support and j represents the potential chemical reactions. Since no chemical reactions are present $r_{j}$ terms are null. Note that the heat exchange mechanisms amongst the different parts of the membrane are included as boundary conditions.

As far as the heat transfer modelling is concerned, no thermal conduction occurs in the axial direction of the membrane reactor; in other words, the Péclet’s number ($Pe=Re\;Pr_{D_{h}}$) is always higher than 1000 for turbulent flow conditions. On the other hand, thermal conduction and convection between the membrane and the feed and permeate streams are considered. The convection heat transfer coefficient is calculated by using the Gnielinski correlation for the Nusselt number in fully developed turbulent and transition flow conditions [23]:

(6) $$Nu_{\infty }=\frac{C_{f}}{8}\frac{\left[Re_{D_{h}}-1000\right]Pr}{1+12.7\;\sqrt{\frac{C_{f}}{8}}\;\left[Pr^{\frac{2}{3}}-1\right]},$$

where,

(7) $$\{\begin{array}{c} 0.5\leq Pr\leq 2000\\ 3000\leq Re_{D_{h}}\leq 10^{6} \end{array}.$$

Laminar conditions are simulated using the heat and mass transport analogy. 

### 3.3. Permeation Model

**Figure 2:**
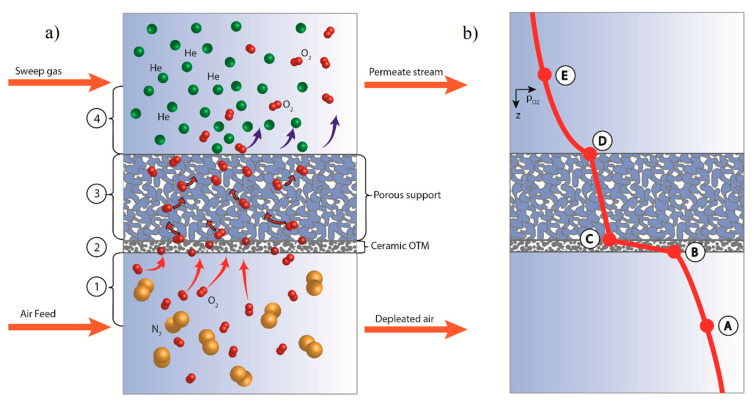
Schematic representation of the membrane module (**a**) and the associated O_2_ partial pressure qualitative profile (**b**).

Each control volume is characterized by an O_2_ partial pressure profile and permeation process, as the one depicted in Figure 2. The main steps of the permeation process and a qualitative profile of the O_2_ partial pressure gradient are highlighted in the *z*-axis direction in a supported oxygen-ion conducting membrane. The permeation mechanism is sustained by means of an oxygen partial pressure gradient between the feed and permeate compartments. 

With reference to the left sketch of Figure 2, four main oxygen transport steps are distinguished in a supported planar membrane:

1. Gas phase diffusion of molecular oxygen from the bulk phase to the membrane surface on the feed side.
2. Diffusion across the ceramic membrane layer; this comprises of the following sub-processes:
  - Oxygen reduction (ORR), which involves: (i) surface exchange reaction on the feed side—chemisorption of the oxygen molecule onto a metal oxide at an oxide-ion vacancy; (ii) dissociation and reduction of O_2_ with the consequent production of oxygen anions; (iii) incorporation of O^2−^ ion into the lattice structure [24].
  - Ionic transport throughout the dense membrane material—single-phase (MIEC) or composite. Simultaneous bulk-diffusion of charged species and small polarons in the bulk phase.
  - Surface-exchange reaction on the permeate interface—oxygen anions recombination to form molecular oxygen and oxygen vacancies in the solid structure
3. Oxygen transport in the porous support structure.
4. Gas diffusion and counter-diffusion of chemical species from the gas bulk to the membrane surface and vice-versa.

#### 3.3.1. Gas Phase Bulk Diffusion

Consider the absolute flux of species i in a mixture along the direction normal to the membrane wall for diffusion in a non-stationary medium:

(8) $$N_{i}=-CD_{ik}\frac{\partial x_{i}}{\partial z}+x_{i}\sum_{i=1}^{n}N_{i},$$

where, $D_{ik}$ is the mutual diffusion coefficient of species i into k and $x_{i}$ is the molar fraction of species i. For the case of an OTM, $i=O_{2}$ and k=mix in air. Considering the conditions on the feed side, the system is characterized by the conservation of the number of moles throughout the whole gas phase, due to the absence of any chemical reaction; therefore, the following holds:

(9) $$\frac{\partial N_{O_{2}}}{\partial z}=0.$$

Moreover, the concentration profile for the oxygen features a decreasing slope ($\partial C_{O_{2}}/\partial z>0$) from the bulk to the membrane wall; on the other hand, the flux of all the other species in the feed mixture is zero, since they are not subjected to any driving force ($N_{mix}=0$). Following these considerations, Equation (8) becomes [23]:

(10) $$N_{O_{2}}=-\frac{CD_{O_{2},mix}}{1-x_{O_{2}}}\frac{\partial x_{O_{2}}}{\partial z},$$

which can be integrated between the bulk phase ($z=\delta_{m}$) and the membrane wall (z=0) through the diffusion layer thickness ($\delta_{m}$), which is taken equal to the hydraulic diameter of the membrane channels [25]. We assume perfect mixing outside the diffusion boundary layer thickness that is, $x_{i}\left(z=\delta_{m}\right)=x_{i}^{b}$. The integrated form results:

(11) $$N_{O_{2}}=\frac{p}{RT}\frac{D_{O_{2},mix}}{D_{h}}ln\frac{1-x_{O_{2}}^{b}}{1-x_{O_{2}}^{w}}\;,$$

where, *b* and *w* represent the conditions of points E, A and D, B respectively on the feed and permeate side. Moreover, the ratio $D_{O_{2},mix}/D_{h}$ is defined as the mass transfer coefficient ($h_{SFM})$ in the stagnant film model [26,27,28], which holds if the velocity of the flow normal to the membrane wall is zero across the whole bulk phase. Within the hypotheses of the stagnant film model, the mass transfer coefficient $\left(h_{SFM}\right)$ is rigorously equal to the overall mass transfer coefficient defined as:

(12) $$h_{m}=-\frac{D_{O_{2},mix}}{C_{O_{2}}^{w}-C_{O_{2}}^{b}}{\frac{\partial C_{O_{2}}}{\partial z}|}_{z=0},$$

where, the mutual diffusion coefficients can be estimated with the Fuller’s method, as described in the kinetic theory [29].

(13) $$D_{ij}=\frac{1.43*10^{-3}\;T^{1.75}}{pM_{ij}^{0.5}{\left[\nu_{i}^{1/3}+\nu_{j}^{1/3}\right]}^{2}},$$

where, $M_{ij}=2{\left(\frac{1}{M_{i}}+\frac{1}{M_{j}}\right)}^{-1}$ and the molecule diffusion volumes ($v_{i}$) can be found in literature [29].

From the definition of the Sherwood number $Sh=h_{m}D_{h}/D_{O_{2},mix}$, Equation (10) applied to the O_2_ flux becomes:

(14) $$N_{O_{2}}=-\frac{Sh_{k}\cdot D_{O_{2},mix}}{D_{h,k}}\cdot \frac{p_{k}}{R\cdot T_{k}}\cdot ln\left(\frac{1-x_{O_{2},b}^{k}}{1-x_{O_{2},w}^{k}}\right),$$

where k represents the feed or permeate side; the binary diffusion coefficient per each component in the mixture is calculated as follows [30]:

(15) $$D_{O_{2},mix}=\sum_{j\neq i}\frac{1-x_{O_{2}}}{\frac{x_{j}}{D_{ij}}}.$$

Moreover, $x_{O_{2},b}^{k}$ and $x_{O_{2},w}^{k}$ represent the oxygen molar fraction in the gas bulk phase and at the membrane wall, respectively. For one-dimensional motion in a straight channel in laminar regime, the Sherwood number is solely dependent on the geometry of the channel. Assuming the heat transfer-mass transfer analogy, it is possible to calculate the Sherwood number with the same correlations as for the Nusselt number in rectangular cross-sectional area channels [23]. For channel width-to-height ratios (*b*/*a*) between 1 and 4, the following interpolation function may be employed.

(16) $$Sh=-0.06475{\left(\frac{b}{a}\right)}^{3}+0.4939{\left(\frac{b}{a}\right)}^{2}-0.5332\left(\frac{b}{a}\right)+3.702.$$

For higher values of b/a ratios, a piecewise linear interpolation of the values in Reference [23] is used. A similar relation can be written for the permeate side.

#### 3.3.2. Solid Phase Bulk Diffusion

As far as the oxygen diffusion mechanism in ionic conductors is concerned, the flux density of the charge carriers is expressed by:

(17) $$j_{k}=-\frac{\sigma_{k}}{z_{k}^{2}F^{2}}\left(\nabla \mu_{k}+z_{k}F\nabla \phi \right),$$

where, $\sigma_{k}$ is the conductivity of the charge carrier k (i.e., electrons or oxygen anions), $z_{k}$ is the number of electrical charges of the carrier k ($z_{O^{2-}}=2$) and μ and ϕ are the chemical and electrostatic potentials across the membrane, respectively. MIECs materials are known to have electronic conductivity $\sigma_{e^{-}}$ orders of magnitude greater than the ionic one $\sigma_{O^{2-}}$.

The coupling of Ohm’s law and Nernst-Einstein equation is used to calculate the electrical conductivity of a material with respect to a defined charge carrier; the first one allows defining the current density as a function of the electrostatic potential gradient in an isotropic medium:

(18) $$i_{k}=-\sigma_{k}\times \nabla \phi =-c_{k}\times z_{k}F\times v_{k}\;,$$

where, $c_{k}$ is the concentration of mobile charge carriers (i.e., electrons, holes) and $v_{k}$ is the velocity of the charge carrier moving in the lattice structure. The latter shall be replaced with the charge carrier drift mobility defined as $u_{k}\;\equiv \;v_{k}/\nabla \phi$.

The Nernst-Einstein equation is used to connect the drift mobility to the electrical conductivity throughout a conductivity-related diffusivity factor $D_{k}$.

(19) $$D_{k}\;=\;RT\frac{u_{k}}{z_{k}F},$$

hence,

(20) $$\sigma_{k}\;=\;z_{k}F\;\times \;c_{k}\;\times \;u_{k}=\;D_{k}c_{k}z_{k}^{2}F^{2}/RT,$$

where, $D_{k}$ can be expressed in an Arrhenius-like formulation $D_{k}=\;D_{k}^{{}^{\circ}}\;exp\left(-\Delta H_{k}/RT\right)$.

Consequently, considering the definition of the chemical potential for an ideal component, $\mu_{k}=\mu_{k}^{{}^{\circ}}+RT\ln C_{k}$ and assuming that the gradient of the electrostatic potential is negligible in comparison to the chemical potential due to the higher electronic conductivity of electrons with respect to oxygen anions, the flux of the charge carrier k can finally be expressed in the form of a Fick’s first law:

(21) $$j_{k}=-D_{k}\frac{\partial C_{k}}{\partial z}.$$

Considering the main charge carrier as the oxygen vacancies in the solid lattice ($k=V_{O}^{••}$) and knowing that $j_{V_{O}^{••}}=-2j_{O_{2}}$, the oxygen flux through the bulk of the solid perovskite lattice structure (step 3) is found and expressed in the preferential direction normal to the membrane wall as:

(22) $$j_{O_{2}}=\frac{D_{k}}{2}\frac{\partial C_{k}}{\partial z}.$$

#### 3.3.3. Superficial Reactions

The overall permeation process and the concentration of the charge carriers on each side of the membrane is also governed by the surface reactions (Equation (23) and Equation (24)), which correspond to steps 2i and 2iii, respectively.

(23) $$\frac{1}{2}O_{2}+V_{O}^{••}\overset{k_{f}/k_{r}}{↔}O_{O}^{X}+2h^{•}$$

(24) $$O_{O}^{X}+2h^{•}\overset{k_{r}/k_{f}}{↔}\frac{1}{2}O_{2}+V_{O}^{••},$$

where, $V_{O}^{••}$ and $h^{•}$ are the charge carriers for the ionic and electronic conduction process respectively and $O_{O}^{X}$ are the oxygen atoms embedded in the lattice structure of the perovskite membrane. Moreover, $k_{f}$ and $k_{r}$ are the forward and reverse reaction rate constants and can be expressed with an Arrhenius-like formulation:

(25) $$k_{f}=k_{f}^{0}exp\left(\frac{E_{act}^{f}}{RT}\right)$$

(26) $$k_{r}=k_{r}^{0}exp\left(\frac{E_{act}^{r}}{RT}\right).$$

Considering the reported reactions as elementary steps in the overall mechanism and applying the law of mass-actions, which states that the *np* product has to be constant at a given temperature, the charge carriers’ concentrations can be related to the oxygen partial pressure, as follows:

(27) $$n=\left[e{}^{\prime }\right]=\sqrt{\frac{1}{k_{eq}}\frac{\left[O_{O}^{X}\right]}{\left[V_{O}^{••}\right]}}p_{O_{2}}^{-1/4}$$

(28) $$p=\left[h^{•}\right]=\sqrt{k_{eq}\frac{\left[V_{O}^{••}\right]}{\left[O_{O}^{X}\right]}}p_{O_{2}}^{1/4},$$

where, $k_{eq}=k_{f}/k_{r}$ is the equilibrium constant of the surface reaction.

Finally, the reaction rate of the two reactions rates, which also correspond to the oxygen flux through the membrane, shall be expressed as:

(29) $$j_{O_{2}}=k_{f}p_{O_{2}}^{{}^{\prime }0.5}\left[V_{O}^{••}\right]{}^{\prime }-k_{r}\left[O_{O}^{X}]{}^{\prime }[h^{•}\right]{}^{\prime }$$

(30) $$j_{O_{2}}=k_{r}\left[O_{O}^{X}]{}^{\prime \prime }[h^{•}\right]{}^{\prime \prime }-k_{f}p_{O_{2}}^{{}^{\prime \prime }0.5}\left[V_{O}^{••}\right]{}^{\prime \prime },$$

where, the apices ${}^{\prime }$ and ${}^{\prime \prime }$ indicate respectively the feed (reduction, surface B in Figure 2) and the permeate (oxidation, surface C) side. The final assumption for the surface reaction model follows the hypothesis of Xu and Thomson 1999 [31], which states that the two surface reactions could be considered as pseudo-zero order, as far as the concentration of oxygen ions and electron vacancies; therefore, the two equations—Equation (29) and Equation (30)—could be considered independent on $\left[h^{•}\right]$ and $\left[O_{O}^{X}\right]$. This assumption, as verified by Hunt et al. 2014 [32], does not contribute to a great variation in the oxygen flux, which is only slightly overestimated in the zero-order equation.

The combination of Equation (22), Equation (29) and Equation (30) allows obtaining a simplified equation for the oxygen flux through a perovskite membrane dependent only upon the bulk diffusion, the surface reaction process and the thickness of the membrane itself.

(31) $$j_{O}{}_{2}=\frac{D_{V_{O}^{••}}k_{r}\left(p_{O_{2}}^{{}^{\prime }0.5}-p_{O_{2}}^{{}^{\prime \prime }0.5}\right)}{2Lk_{f}{\left(p_{O_{2}}^{{}^{\prime }}p_{O_{2}}^{{}^{\prime \prime }}\right)}^{0.5}+D_{V_{O}^{••}}\left(p_{O_{2}}^{{}^{\prime }0.5}+p_{O_{2}}^{{}^{\prime \prime }0.5}\right)}.$$

However, the oxygen transport across membranes characterized by a thin dense layer and operating at high temperature are found to be mostly governed by kinetic processes, whilst the ionic transport resistance within the lattice structure results to be negligible. This assumption was already verified by Chen et al., 1997 [33] and reported in Plazaola et al., 2019 [13], whereby the oxygen permeation becomes partially limited by surface oxygen exchange for thicknesses below 1 mm. The rate limiting step is constituted by the recombination of oxygen ions (O^2−^) that permeate through the membrane lattice to form molecular oxygen on the permeate side surface.

In this scenario, the first term of the denominator in Equation (31) can be neglected and the oxygen flux can be calculated by the following equation:

(32) $$j_{O_{2}}=\frac{k_{r}\cdot \left(\sqrt{p_{O_{2},B}}-\sqrt{p_{O_{2},C}}\right)}{\left(\sqrt{p_{O_{2},B}}+\sqrt{p_{O_{2},C}}\right)},$$

where, $p_{O_{2},B}$ and $p_{O_{2,}C}$ are the oxygen partial pressure on the opposite surfaces of the membrane while *k_r_* is the kinetic constant of the recombination reaction.

#### 3.3.4. Permeation in Porous Media

The model considers the possibility of simulating supported membranes (step #3 in Figure 2). One of the most employed models for the simulation of mass transport in a porous media is the Dusty Gas Model (DGM), which consists of an application of the Maxwell-Stefan diffusion equation [30]. The DGM considers the parallel contribution of three different diffusion mechanism: (i) bulk diffusion (molecule-molecule collision dominated); (ii) Knudsen diffusion (molecule-wall collision dominated); (iii) surface diffusion/micro-pore diffusion (diffusion of adsorbed species along the wall). The model implicit formulation can be written as:

(33) $$-\frac{c_{i}}{RT}\frac{\partial \mu_{i,T}}{\partial z}=\sum_{\begin{array}{c} j=1\\ j\neq i \end{array}}^{n}\frac{\left(c_{j}j_{i}-c_{i}j_{j}\right)}{cD_{ij}^{eff}}+\frac{j_{i}}{D_{Kn,i}^{eff}}\;\left(i=1,2,\ldots ,n\right),$$

where, $c_{i}$ is its concentration and $\mu_{i,T}$ is the chemical potential at a fixed temperature; moreover, $D_{ij}^{eff}$ and $D_{Kn,i}^{eff}$ are the effective binary and Knudsen diffusion coefficients corrected by the ratio of the porosity and tortuosity (ϵ/τ) of the porous structure. The binary diffusion coefficient is estimated through the kinetic theory [29] as already reported in Equation (13). The Knudsen coefficient is equal to:

(34) $$D_{Kn,i}=\frac{2}{3}r_{pore}\sqrt{\frac{8RT}{\pi M_{i}}},$$

where, $r_{pore}$ is the average radius of the pores in the support.

The chemical potential can be expressed as a function of the pressure gradient as:

(35) $$d\mu_{i}=RTdlnx_{i}+{\bar{V}}_{i}dp$$

Finally, introducing the overall flux of the species i expressed by Equation (8) as, $N_{i}=j_{i}+c_{i}v$, Equation (33) becomes:

(36) $$-\frac{p}{RT}\frac{\partial x_{i}}{\partial z}-\frac{x_{i}}{RT}\left(1+\frac{pB_{0}}{\mu D_{Kn,i}^{eff}}\right)\frac{\partial p}{\partial z}=\sum_{\begin{array}{c} j=1\\ j\neq i \end{array}}^{n}\frac{x_{j}N_{i}-x_{i}N_{j}}{D_{ij}^{eff}}-\frac{N_{i}}{D_{Kn,i}^{eff}},$$

where, the velocity in the advective term can be modelled by means of the Darcy’s law for viscous flows [34]:

(37) $$v=-\frac{B_{0}}{\mu }\frac{\partial p}{\partial z}.$$

Considering that the support bulk structure is not characterized by any chemical or electrochemical reactions processes, the number of moles remains the same throughout the whole of its thickness. This hypothesis supports using the Graham’s law which states the absence of a pressure gradient as a consequence of reaction stoichiometry [30].

(38) $$\sum_{i=1}^{n}N_{i}\sqrt{M_{i}}=0\left(\frac{\partial p}{\partial z}=0\right).$$

Finally, considering the methodology reported in Zhu et al. 2005 [35] to develop a direct equation for the DGM, the following simplified oxygen flux formulation is obtained ($i=O_{2}$).

(39) NO2=ϵτ⋅(11DKn+1−pO2DpCDO2j)⋅(cO2D−cO2Ctsupport).

Therefore, at fixed partial pressures on the feed and permeate side (i.e., $p_{A}$ and $p_{E}$), the model employs Equation (14) (for both feed and sweep sides), Equation (32) and Equation (39) and solves the nonlinear equation system employing a modified Powell hybrid algorithm and finite-difference approximation to the Jacobian to obtain $j_{O_{2}}$, $p_{B}$, $p_{C}$ and $p_{D}$.

## 4. Calibration of Permeation Model

### 4.1. Experimental Set-Up

The permeation of asymmetric La_0.6_Sr_0.4_Co_0.2_Fe_0.8_O_3-δ_ (LSCF) membranes and bare porous support are measured at RSE laboratories in the frame of the FP7 European Project GREEN-CC. Disk-shaped samples are manufactured and provided by Forschungszentrum Jülich GmbH (Jülich, Germany). Asymmetric membranes consist of a 30 μm thick dense LSCF layer, supported on a 0.7 mm porous LSCF structure. No catalytic active layer was deposited on the tested samples. The membrane has a circular shape with a diameter of 13.7 mm and its active area is 147.4 mm^2^. The support porosity is estimated by a quantitative image analysis by using a Nikon LUCIA software and it is approximately 0.45.

**Figure 3:**
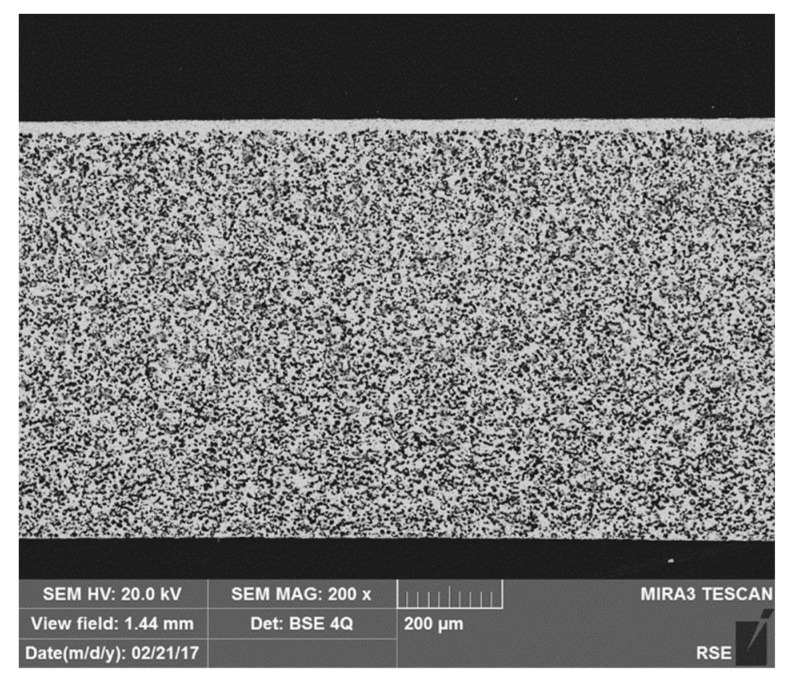
Image of the polished cross section of an asymmetric membrane.

A SEM (Scanning Electron Microscopy) image of the polished cross section of an asymmetric membrane is shown in Figure 3.

**Figure 4:**
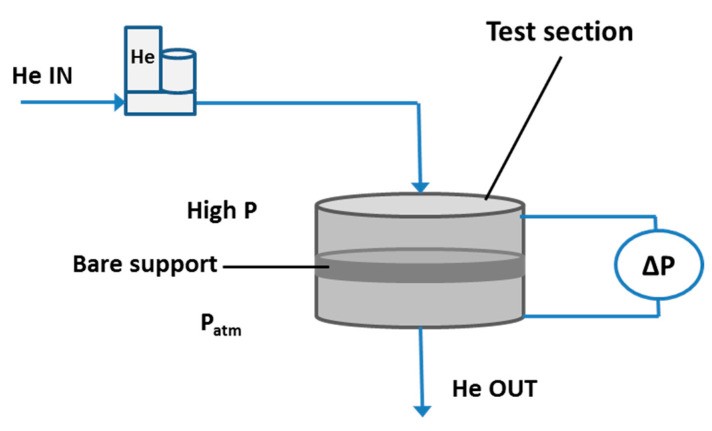
Experimental setup for test on bare support at room temperature.

LSCF bare porous supports, with a thickness of 1 and 2 mm are tested at room temperature, in pure Helium, by feeding a known feed gas flow rate to the test section in the absence of sweep gas and by measuring the pressure difference across the sample, as depicted in Figure 4.

**Figure 5:**
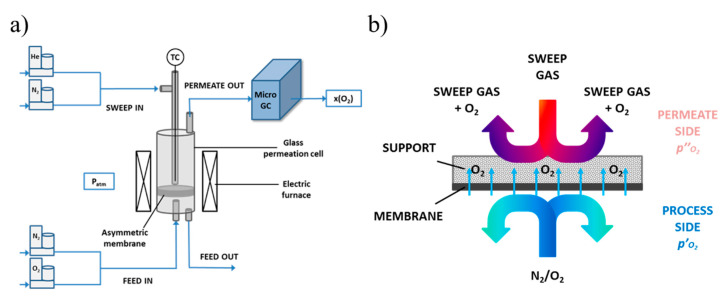
(**a**) Conceptual scheme of the experimental setup for high temperature O_2_ permeation tests; (**b**) configuration of the supported membrane during permeation tests.

High temperature permeation tests are performed on asymmetric membranes in a glass permeation cell, similar to the one described in Reference [36]. The permeation cell is placed in an electric furnace to heat the system to the operating temperature, monitored by a thermocouple in the sweep gas tube (Figure 5a). The gas tightness between the membrane and the permeation cell is ensured at high temperature (approx. 1050 °C) by two pure gold O-rings, on both side of the sample. Tests are carried out in the 700–950 °C temperature range, by feeding air or pure oxygen as process gas on the membrane side and helium or nitrogen as sweep stream on the porous support side. The test configuration is reported in Figure 5b. Both the process gas and the sweep gas are fed at atmospheric pressure and are mass flow controlled—the process gas flow rate is kept constant at 250 NmL min^−1^ and 200 NmL min^−1^, in the case of air and pure oxygen, respectively, while the sweep gas flow rate is varied between 100 and 300 NmL min^−1^.

The oxygen permeation is estimated by measuring the oxygen concentration in the permeate stream by an online micro-Gas Chromatographer (m-GC), while the absence of membrane leakage is ensured by continuously monitoring the nitrogen concentration in the permeate side. The experimental error on the O_2_ flux measure results to be at maximum 4%.

### 4.2. Results of Experimental Tests

**Figure 6:**
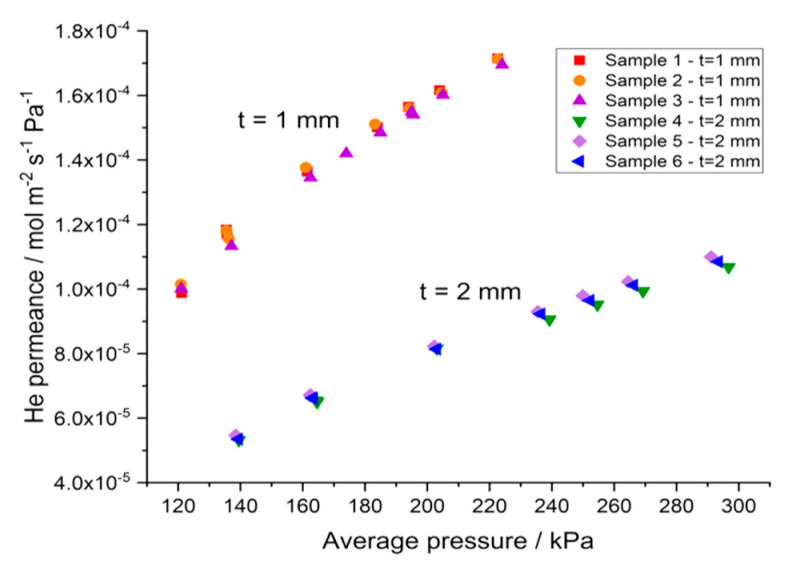
Helium permeance as a function of the average pressure measured across the sample.

The helium permeance of six different bare supports, 1 or 2 mm thick, is determined at room temperature as a function of the average pressure measured across the sample, to verify the homogeneity of the samples’ porosity. The comparison between results obtained on samples with different thickness, is reported in Figure 6 as a function of the average pressure [37].

As expected, the He permeance measured on supports with a thickness of 2 mm is about two times lower than the permeance measured on thinner supports (1 mm), therefore the reduction of the He permeance is proportional with the increase of the support thickness.

**Figure 7:**
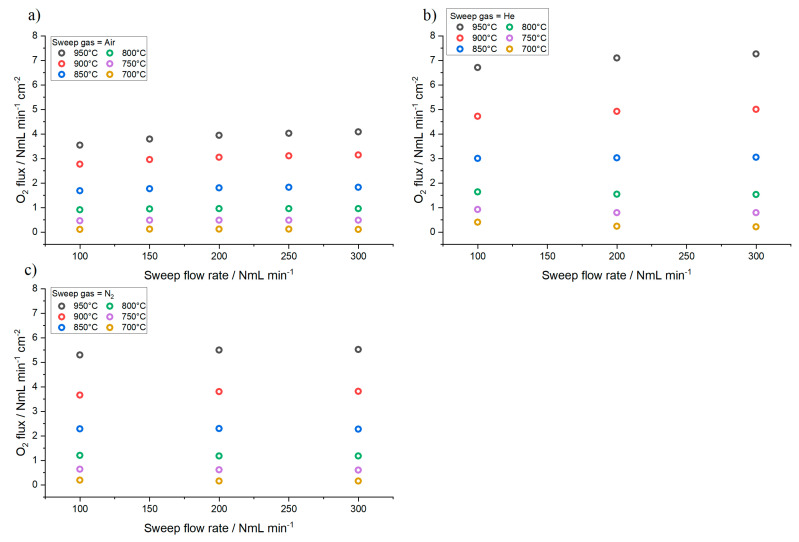
Measured oxygen flux as a function of the sweep gas flow rate for different temperature. Permeation tests performed by: (**a**) feeding air as process gas (N_2_ = 197.5 NmL min^−1^, O_2_ = 52.5 NmL min^−1^) and helium as sweep; (**b**) pure oxygen as process gas (O_2_ = 200 NmL min^−1^) and helium as sweep; (**c**) or nitrogen as sweep gas.

Oxygen permeation tests are performed at high temperature on an asymmetric membrane by feeding synthetic air (with a constant flow rate of N_2_ = 197.5 NmL min^−1^, O_2_ = 52.5 NmL min^−1^) or pure oxygen as process gas (with a constant flow rate of 200 NmL min^−1^) and helium or nitrogen as sweep gas, by varying the flow rate in the range 100–300 NmL min^−1^. Results are shown in Figure 7, in terms of oxygen flux as a function of the sweep gas flow rate for different temperature [38].

As predicted by Equation (31), the oxygen flux increases with the temperature. By feeding pure oxygen instead of air as process gas, an improvement of the oxygen permeation is observed, due to the increase of the oxygen partial pressure in the process side $\left({p^{\prime }}_{O_{2}}\right)$ and, consequently, of the process driving force.

**Figure 8:**
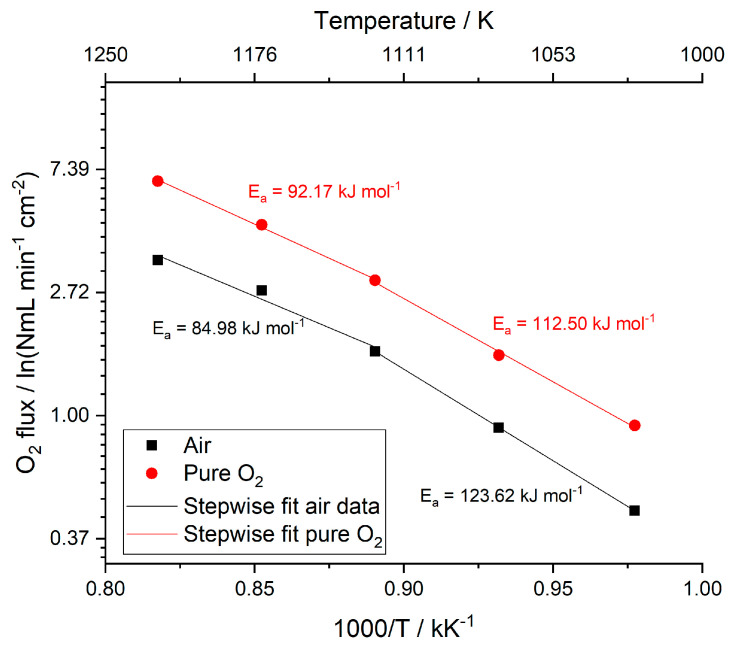
Oxygen flux as a function of the inverse of the temperature. Permeation tests performed by feeding air or pure oxygen as process gas and helium as sweep gas, with a flow rate of 100 NmL min^−1^ between 750 °C and 950 °C.

The membrane permeation can be also described by an Arrhenius-type correlation. As shown in Figure 8, in the range 950–750 °C a different Arrhenius behavior can be observed, which suggests a variation in the membrane permeation mechanism as a function of the temperature. At high temperature, the bulk diffusion through the membrane dense layer mainly controls the permeation, while at lower temperature the oxygen surface exchange is predominant [12,39]. The estimated activation energy values are in accordance with the results reported by Serra et al. 2013 [12].

The oxygen flux through the membrane increases with rising temperature, both in the presence of helium and nitrogen. At high temperature (850 °C–900 °C), oxygen flux is slightly influenced by the increase of sweep gas flow rate due to the higher oxygen dilution in the permeate and by the increase of the process driving force. On the other hand, at lower temperatures permeation is almost constant in the investigated sweep gas flow rate range. Moreover, the oxygen flux is slightly higher when helium is used as sweep gas, instead of nitrogen.

In summary, the experimental data are available at 950 °C, 900 °C and 850 °C and atmospheric pressure. The feed and permeate streams are changed in the following conditions:

▪ air feed (200 NL min^−1^) and helium as sweep gas (100–300 NL min^−1^),
▪ oxygen feed (200 NL min^−1^) and helium as sweep gas (100–300 NL min^−1^),
▪ oxygen feed (200 NL min^−1^) and nitrogen as sweep gas (100–300 NL min^−1^).

### 4.3. Support Calibration

Given the geometric characteristics of the model presented, it cannot be directly applied to evaluate the permeation of the circular membrane as the one used for the tests. Introducing the actual axisymmetric flow field occurring in the real disk-shaped membrane setup in the presented model requires the application of CFD simulations that is beyond the scope of this study. In order calibrate the main parameters of the model against the experimental test, the permeation model (Equation (1) to Equation (32)) is applied to evaluate the resulting O_2_ flux considering the thermodynamic and chemical variables averaged between inlet and outlet on each side of the circular membrane in a single discretization step.

To calibrate the unknown parameters of the support ($r_{pore}$ and τ), the set of permeance data for helium are considered (see Figure 5). At the highest end of the measured flow rates range, the flow regime results turbulent; therefore, Equation (39) is not able to completely describe the mass transfer phenomena occurring in the porous support, because it is developed for laminar diffusion regime. Hence, a mixed laminar-turbulent diffusion semi-empirical model is employed [40].

(40) $$\Delta p=J\cdot t\left[\frac{\mu }{\alpha }+\frac{\rho }{\beta }J\right],$$

where,

- *J* [m^3^ s^−1^ m^−2^] is the volumetric flux across the porous structure.
- *t* [m] is the support thickness.
- *μ* [Pa s] is the dynamic viscosity.
- *α* [m^2^] is a viscosity coefficient which represents the Δ*p* losses due to diffusivity effects. This is a calibration parameter.
- *ρ* [kg m^−3^] is the average density.
- *β* [m] is a momentum coefficient which represents the Δ*p* losses due to turbulent effects. This is a calibration parameter.

**Figure 9:**
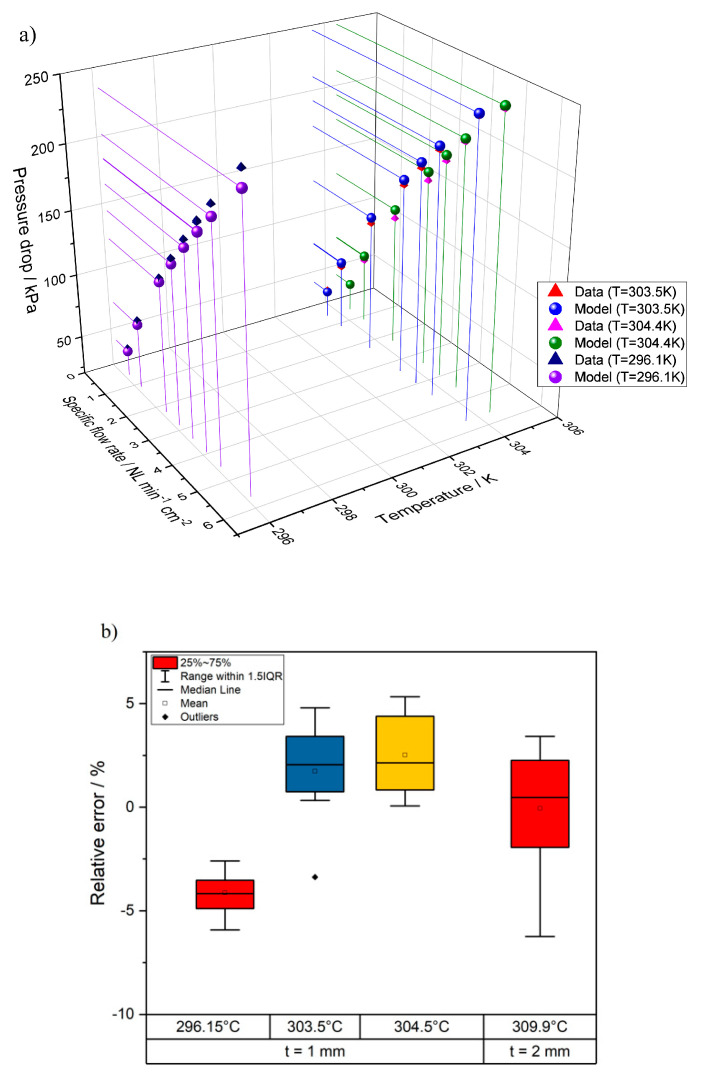
Summary of the support calibration procedure: (**a**) comparison between measured pressure drop data and model predictions for *t* = 1 mm; (**b**) relative error between data and model for both *t* = 1 mm and *t* = 2 mm.

The parameters *α* and *β* are calibrated against the support permeance experimental data minimizing the sum of the root mean square of the relative difference between the model and the measured data. The calibrated values in the considered operating range are *α* = 4.27 × 10^−8^ m^2^, *β* = 3.41 × 10^−9^ m. The average error results to be 3.14% with a maximum error comprised between −6.24% and 5.33%. The result of the calibration is represented in Figure 9.

Subsequently, the pressure drop obtained from the semi-empirical model are employed to calibrate the physical model in Equation (39) for the DGM in the laminar flow regime (1–12 NmL min^−1^-cm^2^). Being the experimental data relative to the permeation of a pure species (i.e., He) across the porous support, Equation (39) reduces to a simplified form which depends solely upon the Knudsen diffusivity $D_{Kn}$ and not on the molecular diffusivity in a binary mixture $D_{He,j}$. Consequently, knowing the geometry of the support—thickness 1.0 and 2.0 mm—and its porosity 0.45, the only unknown characteristics is the ratio between the pore radius and the tortuosity, that is, $r_{pore}/\tau$. The DGM is calibrated in the flow regime of interest for laminar mass transport diffusion in a porous media by finding the $r_{pore}/\tau$ ratio which minimises the sum of the root mean square of the relative errors between the model and the measured data. The resulting pore radius-to-tortuosity ratio $r_{pore}/\tau$ is 0.581 μm. As far as the value of tortuosity of the porous media, a value equal to τ=1.765 is taken, as results from applying the Bruggeman equation $\tau =\frac{{\left(1+\epsilon \right)}^{2}}{\epsilon {\left(1+\epsilon \right)}^{2}+4\epsilon^{2}\left(1-\epsilon \right)}$, as reported in Kong et al., 2015 [41]; the resulting value of tortuosity is also in line with the simulations performed via microstructure reconstruction by Joos et al., 2011 [42]. The resulting *r_pore_* is therefore 1.03 μm.

### 4.4. Supported Membrane Calibration

To calibrate the supported membrane model, the kinetic coefficients ($k_{r}^{0}$, $E_{r}^{0}$) of the Arrhenius-like equation for the kinetic constant in Equation (32) are calibrated by minimizing the mean squared deviation between the experimental and calculated oxygen flux in a temperature range between 850–950 °C. The minimization procedure employs the genetic algorithm solver available with Microsoft Excel. The calibration is performed by considering a planar membrane of the same active area (147.4 mm^2^) and porous support thickness (i.e., 0.7 mm) of the experiments.

A rectangular geometry with co-current flows is considered. Length is assumed equal to the radius of the circular membrane (6.85 mm) tested. The resulting channel width is 21.5 mm. Since the width-to-height ratio (*b*/*a*) for both sweep and feed channels is impossible to define in the experimental set-up, this parameter is treated as the third calibration parameter, constrained in a range 1–∞. Moreover, the assumption of negligible pressure losses is employed for the calibration case.

In summary, the calibration of the supported membrane consists of a constrained optimization with the following objective function, variables, and limits:

***Objective Function:***

$$min{\left(\sum {\left(\frac{J_{O_{2}}^{Data}-J_{O_{2}}^{Model}}{J_{O_{2}}^{Data}}\right)}^{2}\right)}^{0.5}$$

***Variables:***

$$b/a_{sweep},\;b/a_{feed}\;k_{r}^{0},\;E_{r}^{0}$$

***Constraints:***

$$1.0<\frac{b}{a}<\infty$$

The calibrated parameters for the supported membrane result:

- $b/a_{sweep}=\;3.01$
- $b/a_{feed}=\;1.00$
- $k_{r}^{0}=83.61\;\mathrm{kmol}/m^{2}s$
- $E_{r}^{0}=138.36\;\mathrm{kJ}/\mathrm{mol}$

**Figure 10:**
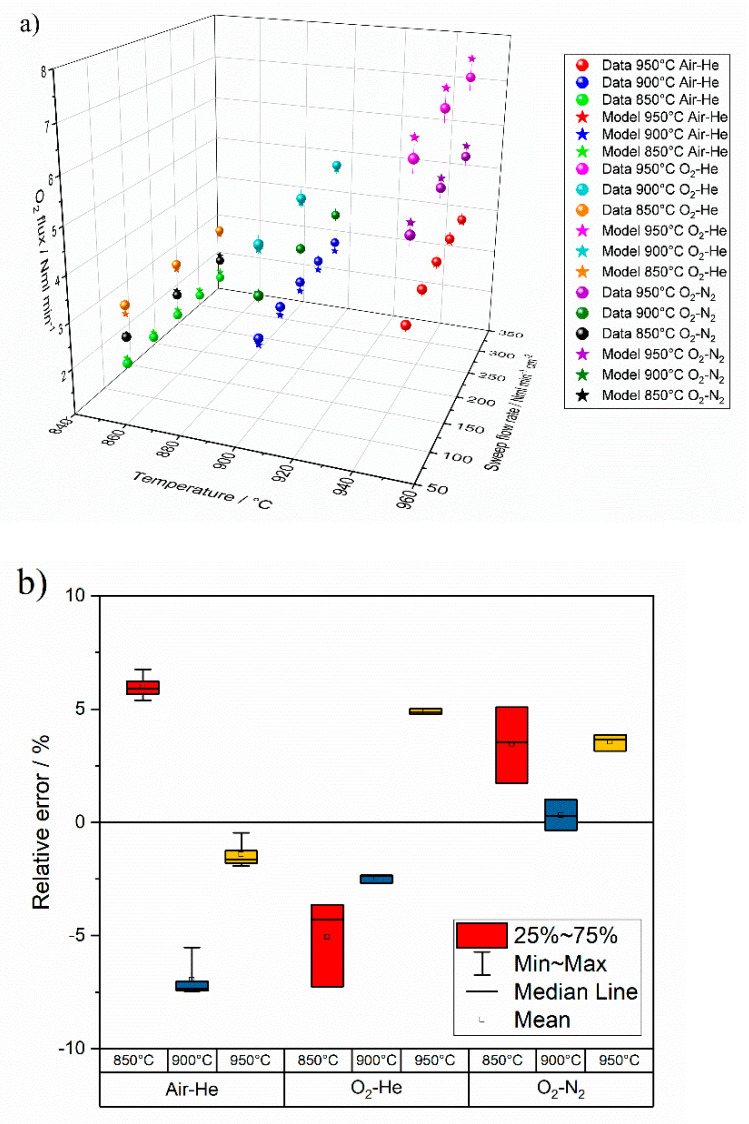
Summary of supported membrane calibration procedure. (**a**) comparison of measured O_2_ flux data and model predictions at different operating temperature and sweep flow rate; (**b**) relative error between measured data and model.

The average root mean square of the error on the experimental data available is 3.99%, thus considered acceptable throughout the whole operating range. Figure 10 reports the results of the calibration procedure in terms of comparison between modelling and experimental data (Figure 10a) and prediction error (Figure 10b). The values of permeation activation energies support the claim that the main process is governed by oxygen recombination reaction on the permeate side.

## 5. Heat and Mass Balance of Glass Melting Furnace

In this work, we apply the OTM technology to a glass melting furnace. The work by Sardeshpande et al. [5] proposed a model to achieve the minimum specific energy consumption in glass furnaces. Based on field analyses and experimentations in the glass industry, the paper provides the design and operating parameters of a case study representing the current state of the art of glass furnaces. The furnace’s capacity and draw are fixed at 100 ton day^−1^ and 90 ton day^−1^, respectively, whereas the cullet fraction is set at 40%. Comparing with Beerkens [6], it is possible to appreciate that it is a very efficient benchmark, even considering the actual cullet (40% vs. 50%), as the energy input amounts to 3833 kJ kg^−1^.

Starting from the work by Sardeshpande et al. [5], heat and mass balances are estimated with GS, a computer code in-house developed in the past years at the Department of Energy of Politecnico di Milano [43]. The code is a powerful and flexible tool that can accurately predict the performance of a wide variety of chemical processes and power generation systems. GS was originally designed to assess the performance of gas-steam cycles for power production [44] and has been progressively developed and improved to calculate complex systems including coal gasification, chemical reactors, fuel cells and essentially all the processes present in advanced plants for power generation [45,46,47,48]. When addressing the new oxy-fuel furnace solutions, the detailed membrane model, as previously described, is employed to propose a reactor design which could fit the process operating conditions derived from GS.

This section presents four cases of glass melting furnaces: Case A: air-blown reference furnace; Case B: oxy-fuel furnace with heat recovery; Case C: oxy-fuel furnace with integrated 3-end OTM module; Case D: oxy-fuel furnace with integrated 4-end OTM module. The results of mass and energy balances in terms of mass flow rates, temperatures and compositions are reported in Appendix A, along with the calculation assumptions for the fluid machinery. The fuel input to the furnace is always natural gas with a lower heating value (LHV) of 46.48 MJ kg^−1^ (see Appendix A for the composition).

### 5.1. Air-Blown Reference Case

**Figure 11:**
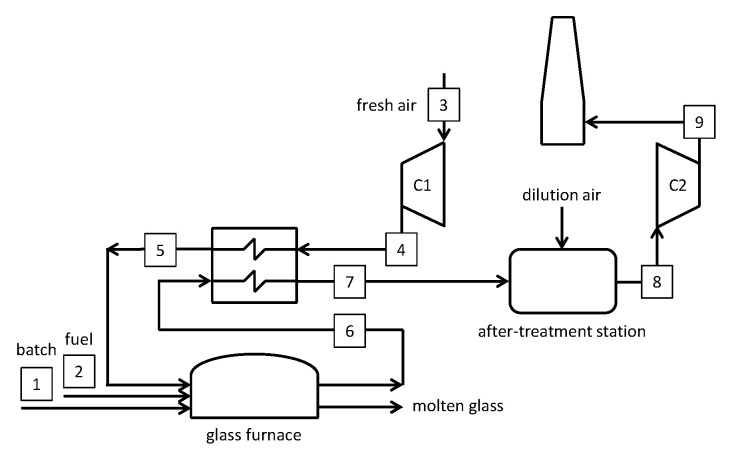
Schematic of the air-blown reference furnace.

The first case considered is the benchmark, according to the work by Sardeshpande et al. [5]. Figure 11 shows a simplified lay-out of the air-blown system and Table A1 in Appendix A reports the main streams’ thermochemical conditions as resulting from the mass and energy balances. The combustion air flow rate is set to obtain an O_2_ content of 2% for the in the gas exiting the furnace [5].

A larger mass flow rate can be observed at station #7 compared to station #6 because of air infiltration, which is calculated according to an O_2_ content at the regenerator output of 8% [5]. In the case of no air infiltration, a temperature of 610.3 °C would be calculated instead of 361.8 °C, considering the heat losses from the regenerator. The after-treatment station cools down the gas at the furnace exit to 150 °C by diluting with ambient air. The resulting stream is de-dusted and scrubbed before being blown to the chimney (stream #8 is significantly larger than stream #7).

### 5.2. Oxyfuel Case with VSA Unit

Compared to a similar air-blown case, Beerkens and Muysenberg [49] reported that an efficient oxy-fuel glass melting furnace requires ~16.7% less fuel; however, such saving does not take into account the corresponding energy consumption required for oxygen production. Therefore, with reference to the calculations in Case A, the input fuel flow rate is tuned to maintain constant the thermal heat loss of the furnace for a fixed production capacity (90 ton day^−1^) [49]. Without varying the temperature of the gas leaving the furnace, the heat loss is reduced by 23.5%, compared to Case A.

The overall energy consumption for an oxy-fuel glass melting furnace is approximately equal to the one in case of air combustion [7]. Beerkens [7] assumed 0.375–0.4 kWh for the electricity consumption per Nm^3^ of pure oxygen generation. Based on an electric plant efficiency of 40%, ~3400 kJ Nm^−3^ are assumed for the primary energy consumption. The new oxy-fuel case results in almost equal total fuel consumption (exactly 1.1% higher) compared to Case A, fully consistent with the technical literature [7].

**Figure 12:**
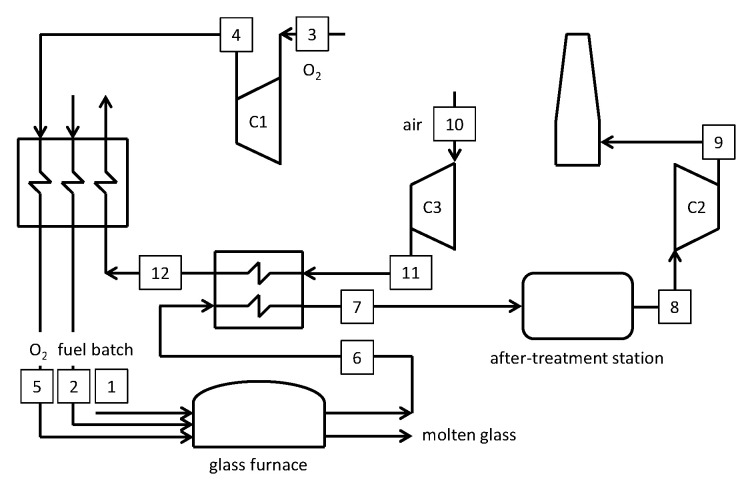
Schematic of the oxy-fuel furnace with heat recovery.

Non-regenerative furnaces may be advantageous from an energy viewpoint compared to conventional regenerative furnaces because the exit temperatures are higher and allow for a better heat recovery section. Inspired by the HotOxyGlass project [50], Figure 12 shows the schematic of an oxy-fuel glass furnace where air (#11) is pre-heated in a recuperator by the high-temperature gas exiting the furnace (#6). This hot air stream (#12) is then directed to other heat exchangers for pre-heating both the oxidizer resulting from a VSA unit and the natural gas. In this new case a further reduction in natural gas consumption is achieved, with an overall fuel saving of 23.8% compared to Case A. Although such a fuel saving does not include the primary energy related to the oxygen production, this result is promising since the HotOxyGlass project [50] declared a saving of 25% ± 2%. After including the necessary energy contribution for oxygen production, the final saving will be lower.

As reported in Table A2 and differently from the previous case, streams #6 and #7 are equal in flow rate. However, a larger air dilution in the after-treatment station is required in this case since the temperature of stream #7 is higher compared Case A.

### 5.3. Oxyfuel Cases with Integrated OTM Systems

Two innovative cases are investigated in this paper, both considering a membrane-based system for the separation of oxygen from a hot air stream. The layouts are designed to self-produce the oxygen required for the combustion in the glass melting furnace and the electricity for all auxiliary loads. The proposed cases consider 3- and 4-end membrane systems, without and with sweep stream fed to permeate side.

**Figure 13:**
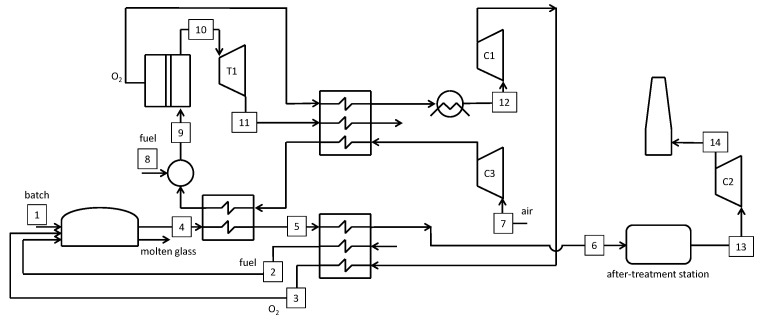
Schematic of the oxy-fuel furnace with integrated 3-end OTM module.

A first layout for the advanced glass melting furnace is schematized in Figure 13 (Case C). Details of the main streams are reported in Table A3 in the Appendix A. Air (#7) is delivered by a compressor and pre-heated up to 600 °C through a recuperative heat exchanger by the hot gas from the turbine T1 (#11) and the high-temperature oxygen from the membrane. Subsequently, air is heated up to ~700°C in another heat exchanger by the high-temperature gas (#4) at the furnace exit. After the combustion with natural gas to rise the temperature up to 950 °C, the stream (#9) enters the membrane system at 4 bar at high (>18 mol.%) O_2_ content. The OTM separates 50 wt.% of the oxygen in the feed stream on the permeate side at 0.25 bar, which is then cooled down to 40 °C (#12). Ultimately, the oxidizer is delivered to the furnace by a vacuum pump (C1) through a recuperative heat exchanger, where it is pre-heated (up to 550 °C) together with the natural gas required at the burners.

The results reported in Table A3 are calculated with the hypothesis of no air infiltration in the sub-atmospheric pressure zones of the plant. Air infiltration in permeate side of the membrane would affect the temperature profiles of the multi-flow heat exchanger downstream turbine T1, as well as the temperatures at stations #5 and #6. However, a minimum ΔT_approach_ ≈ 50 °C is always verified from the calculations, hence guaranteeing an ample operative margin in case some air infiltrations occur. The difference in flow rates #6 and #13 is justified by air dilution in the after-treatment station, as in the previous cases.

It is found that the new oxy-combustion furnace consumes 291.6 kg h^−1^ of fuel. This amount is greater than the fuel demand of Case B due to the fuel required to rise the temperature of the feed stream to the membrane-based system. Nevertheless, the fuel demand is reduced by ~6% compared to Case A.

**Figure 14:**
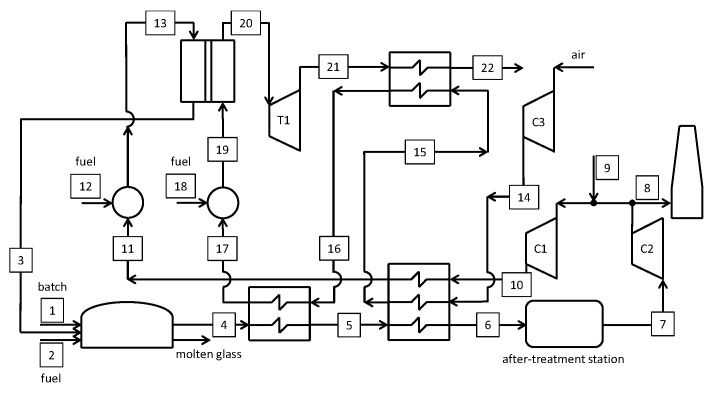
Schematic of the oxy-fuel furnace with integrated 4-end OTM module.

Another layout for the advanced glass melting furnace integrated with the OTM technology is proposed in Figure 14 (Case D). Here we use of a 4-end membrane system with sweep stream fed to the permeate compartment. Differently from Case C, a sweep stream is provided to the membrane by partially recycling the gas exiting the after-treatment station. It is worth noting that the sweep gas (stream #13 in Figure 14) is characterized by a high CO_2_ content. In these conditions, some authors report an increased degradation and performance loss of the LSCF membrane due to segregation of Sr in the form of SrCO_3_ [51]. Other experimental studies suggest the possibility of increased oxygen permeation flux for increasing CO_2_ concentrations in the sweep gas at operating temperatures of 1000 °C [12]. Within the framework of this study, we neglect any effect that CO_2_ might have on the permeation performance of the membrane, confident that the methodology proposed here is equally valid for other types of materials, like NiFe_2_O_4_-Ce_0.8_Tb_0.2_O_2-δ_ (NFO-CTO) that are less susceptible to CO_2_ influence. 

The 4-end case reproduces a solution that is intermediate between the air-blown case and the one with oxygen only. With reference to Figure 14, Case D results much more complex compared to Case C.

Similarly to Case C, it is necessary to raise the temperature of stream #19 up to 950 °C at the inlet of the OTM system, as seen in Table A4.

A sweep stream (#13) enters the permeate side of the membrane system and exits with a fixed O_2_ content (50 mol.%). The gas exiting the glass melting furnace, downstream of the after-treatment station, is partly vented and partly recirculated to the membrane, after mixing with air (#9). Consequently, the O_2_ content in stream #11 is sufficient for raising the temperature of the sweep stream (#13) up to 950 °C by catalytic combustion. Based on the results in Table A4, air flow rate #9 is negligible compared to the exhaust gas recirculation (#3).

### 5.4. Energy Assessment

An overview of the cases presented in the previous paragraphs is reported in Table 1, which details the heat fluxes entering and exiting the glass furnace. The heat in the glass is always the same a fixed production of 90 ton day^−1^ (1628.1 kW), so the fuel reduction for cases B to D is very interesting compared to the reference case. The heat recovery for cases B to D is clearly lower compared to Case A but heat losses are reduced. According to the previous description, Case C, more than Case D, improves Case B from an energy efficiency viewpoint. Heat losses in the flue gases are significantly reduced by transferring heat to the compressed air stream. On the other hand, the heat recovered and recycled into the furnace is lower in Case C compared to Case B, because the oxygen stream delivered by the vacuum pump is already at ~250 °C. When considering Case D, additional fuel is introduced as stream #12 in Figure 14, necessary to raise the temperature of the sweep stream up to 950 °C. Moreover, the heat supplied by O_2_ separation is higher for Case D compared to Case C, where the oxidant is cooled (stream #12 in Figure 13) prior to be delivered by the vacuum pump. As detailed in Table 1, the heat transferred to the micro-gas turbine cycle in Case D is higher than in Case C.

An overview of the energy consumption results of the four cases is reported in Table 2. Details about calculation assumptions for all balance of plant components are reported in Appendix A. The net power from the micro-gas turbine cycle is a system output ad is reported as negative. The overall electric power consumption and production is converted into an equivalent fuel figure, considering an electric conversion of 50% and a LHV of the fuel equal to 46.48 MJ kg^−1^. With reference to the primary fuel results, it is possible to realize the superiority of Case C, from an energy saving point of view. Indeed, Case D does not seem to be more attractive than Case B, owing to the complexity of the deeply integrated system.

### 5.5. Design and Performance of the Integrated OTMs

The design of OTM module for the system configuration with a 3-end OTM and 4-end OTM membrane (i.e., cases C and D) are summarized. For the sweep and non-sweep membrane modules, the flow-field configuration is respectively planar counter-flow and planar co-flow. Each channel geometry is characterized by a fixed width (i.e., 21.5 mm) and a fixed height/width ratio on both feed and sweep side, *b*/*a* = 1.0 and *b*/*a* = 3.01, respectively. Moreover, a mechanical support structure of semi-thickness equal to 2.5 mm on every side of each channel forms the flow-field geometry. This structure does not influence the fluid-dynamic performance of the channels, but it is considered for the calculation of the final module volume. The membrane and support structure and transport parameters are equal to the calibration case. Moreover, each membrane layer is characterized by 20 equal adjacent channels and each stack is made up of 50 layers. This fixes the width and the height of each stack to 0.48 m and 1.59 m, respectively.

The number and length of the membrane channels must be calculated to find the overall dimensions of the membrane module (i.e., membrane area and volume). These two design parameters influence the fluid-dynamic performance, especially in terms of pressure losses. The membrane area is the main input data for the economic analysis. 

**Figure 15:**
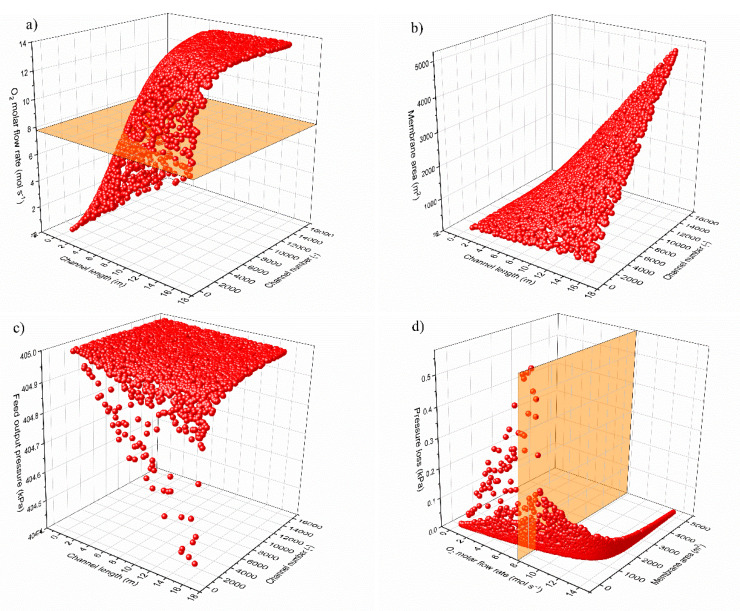
Results of the Monte Carlo simulation (3000 runs) to find the optimal membrane module design for the no-sweep case at 4 bar and SF = 40%. (**a**) oxygen molar flow rate across the membrane as a function of channel length and number of channels; (**b**) total membrane area as a function of channel length and number of channels; (**c**) feed output pressure as a function of channel length and number of channels; (**d**) representation of the optimal design point found at minimum pressure loss and minimum membrane area for a specific value of oxygen flow rate.

A Monte Carlo simulation is used to explicit the functional dependency of the membrane area and the outlet pressure to the number and length of the membrane channels. For a fixed value of oxygen molar flow rate, there are multiple combinations of channel length and number of channels in a membrane module that provide the design constraint, as depicted in Figure 15a. These couples of values are characterized by different values of membrane area and overall pressure loss. Therefore, the objective function of the design optimization is the minimization of the product of the membrane area and the pressure loss. Figure 15b,c represent the functional dependence of the total membrane area and the pressure loss with the two design parameters (channel length and number of channels). Both the membrane area and the overall pressure loss find their minimum with the minimum channel length. 

To find a physical solution, a minimum channel length is fixed equal to at least the stack width (0.48 m). With these assumptions, the best design is found, as depicted in Figure 15d for the specific conditions of a 3-end membrane operating at 4 bar and with a SF = 40%.

The summary of the geometric characteristics for all considered scenarios is reported in Table 3

## 6. Economic Assessment

This section summaries the economic benefits of oxy-fuel glass melting furnaces when oxygen production occurs by means of OTM technology instead of conventional solutions. We focus the comparison between Case B and Case C.

An investment costs (*C*) of 5 M€ and 1 M€ are assumed for a plant capacity (*Q*) of 2000 Nm^3^/h and 200 Nm^3^/h, respectively. Assuming an exponential trend in investment cost as a function of the capacity ratio, that is, *C* = *C*_0_·(*Q*/*Q*_0_)*^f^*, an exponential factor *f* = 0.7 can be inferred from the previous values. Considering that Case B requires around 630 Nm^3^ h^−1^ of pure oxygen, it is possible to estimate 2228.6 k€ as the total cost of the plant for oxygen production via conventional technology. In addition, an energy consumption of 0.4 kWh per Nm^3^ of pure oxygen is assumed.

Referring to Case C, component costs are more thoroughly broken down. We make reference to the methodology proposed by Galanti and Massardo [52] for a quick estimation of these components costs. The direct material costs for the micro-gas turbine system amounts to 491 k€ (as a reference, a cost of 210 k€ for a 200 kW micro-gas turbine is considered). This cost is increased by 30% to take customization into account and further 65.6 k€ are considered for additional recuperative heat exchangers and oxygen vacuum pumping equipment. The sum of such direct material costs is then increased to include EPC services (32%), construction costs (20%) and other costs (8%), resulting in ~1126 k€.

A specific cost of 1900 €/m^2^ is assumed for the OTM module [53]. Based on the calculated membrane area (484.16 m^2^), the cost for the membranes results ~920 k€, plus 10% as the estimated cost for the containing vessel. The resulting direct material costs for the membrane system is then increased to include EPC services (16%), construction (10%) and other costs (8%). The total cost for the membrane system is ~1356 k€. Ultimately the overall Case C system cost results 2482.1 k€.

Additional 93.8 k€ are considered in Case B for recuperative heat exchangers and blowers, not present in Case C’s layout. Such direct material costs ultimately come to 150.1 k€ as the total costs for additional plant engineering.

The total plant costs of the two compared technologies, excepting the glass melting furnace and the after-treatment station, do not seem to differ significantly. 

The additional following economic assumptions are made: 10-year plant life, 8% discount rate, energy valued at the prices of the Italian market (0.79 € Nm^−3^ for natural gas, 0.159 € kWh^−1^ for electricity) and 8000 h of plant operation per year. Economic results are reported in Table 4 for the two cases, considering that the discounted net present value of 10-unit payments equals 6.71 according to the assumed rates. No maintenance costs are considered for the sake of simplicity, even though the membranes could be subject to fouling as well as occlusion. In general, the more complex the system, the greater the maintenance costs.

The energy superiority highlighted in Table 2 for the OTM-based oxy-fuel furnace is further remarked by the results in Table 4. Compared to Case B, the solution proposed in this paper is ~1.8 M€ cheaper on a total cost basis. Further saving can be calculated in case of larger scale production. In this case, after reducing the cost for the membrane down to 1050 € m^−2^ [53], the cost of plant in Table 2 can be revised as 1875.5 k€. This revision leads to possible economic saving of ~2.4 M€ compared to Case B. Indeed, these figures are based on a long-term stability of the membranes, which has not been secured. Therefore, the discounted total costs in Table 4 could be revised in case one or even two membrane stack replacements are considered. Based on such event, Case C would result in a final cost of 17.11 M€ or 18.47 M€, if one or two membrane system replacements are necessary, respectively. Ultimately, switching to the scenario with lower cost of membrane (1050 € m^−2^), the total discounted costs for Case C result 15.91 M€ and 16.66 M€, highlighting again the promising economic results of the OTM technology.

## 7. Conclusions

Oxygen transport membranes are studied in this work for application into the glass production industrial sector. Oxygen membranes are identified as a potential solution for low specific energy consumption and high energy density solution for oxygen separation, in competition with other more conventional systems, such as pressure or vacuum swing adsorption and cryogenic separation.

A detailed mathematical modelling of the physical and chemical processes in an oxygen transport membrane is presented to predict its thermodynamic performance when integrated into a more complex system. Starting from an air-blown reference benchmark case, the mass and energy balances of glass melting furnaces are reproduced for three oxy-fuel furnaces: the first one with oxygen produced by a vacuum swing adsorption unit and the other two where the oxygen is separated from a hot air stream in 3- and 4-end membrane-based systems integrated with a micro-gas turbine.

The assessment of the primary fuel demand of the furnaces highlights the superiority of the oxy-fuel technology compared to the air-blown reference case. The energy demand results to be reduced respectively by ~12% for the VSA based plant and ~22% for the 3-end membrane system. The preliminary economic analysis shows the latter one is more convenient in terms of discounted value than the oxy-fuel solution based on a VSA system. On the other hand, calculations of a 4-end membrane system do not suggest similar conclusions, owing to a more complex and capital-intensive system.

## Figures and Tables

**Table 1:** Details of heat fluxes entering and exiting the glass furnace.

	A	B	C	D
Fuel energy introduced in the furnace, kW_th_	3992.7	3043.5	3026.6	3368.4
Heat carried in the glass, kW_th_	1628.1	1628.1	1628.1	1628.1
Heat recovered and recycled into the furnace, kW_th_	1542.7	218	153.6	251.5
Heat supply from O_2_ separation, kW_th_	-	-	55.4	271.4
Heat introduced by supplementary fuel, kW_th_	-	-	-	121.0
Heat loss in the flue gas, kW_th_	910.4	475.2	217.3	194.0
Other heat losses, kW_th_	1464.6	945.9	945.9	1091.7
Heat transferred to the micro-gas turbine cycle, kW_th_	-	-	290.9	860.4

A: reference case. B: oxy-fuel case with heat recovery. C: oxy-fuel case with 3-end OTM system. D: oxy-fuel case with 4-end OTM system.

**Table 2:** Details of fuel and electricity consumptions for the examined cases.

	A	B	C	D
Fuel to the glass melting furnace, kW_th_	3992.7	3043.5	3043.5	3368.4
Electric power duty due to auxiliaries, kW_el_	105.1	47.3	77.1	8.4
Electric power duty for O_2_ production, kW_el_	-	275.2	-	
Electric power from the micro-gas turbine, kW_el_	-	-	−319.6	−374.2
Overall electric power, kW_el_	105.1	322.5	−242.5	−365.8
Equivalent fuel, kg/h	16.3	50.0	−37.6	−56.7
Additional fuel, kg/h	-	-	57.1	75.8
Primary fuel, kW_th_	4202.9	3688.5	3280	3616

A: reference case. B: oxy-fuel case with heat recovery. C: oxy-fuel case with 3-end OTM system. D: oxy-fuel case with 4-end OTM system.

**Table 3:** Design characteristic summary for the membrane modules in Case C and Case D.

		No Sweep (3-end) OTM			Sweep (4-end) OTM
Split fraction	%	40%	50%	60%	50%
O_2_ Molar flow rate	mol s^−1^	7.76			8.80
channel width	mm	21.5	21.5	21.5	21.5
wall thick	mm	2.5	2.5	2.5	2.5
channel per layer	-	20	20	20	20
membrane width	m	0.480	0.480	0.480	0.480
layers per stack	-	50	50	50	50
b/a_feed_	-	1.00	1.00	1.00	1.00
b/a_perm_	-	3.01	3.01	3.01	3.01
membrane thickness	μm	30.00	30.00	30.00	30.00
support thickness	mm	0.70	0.70	0.70	0.70
membrane height	m	0.032	0.032	0.032	0.032
membrane per stack	-	50	50	50	50
stack height	m	1.590	1.590	1.590	1.590
channel length	m	0.480	0.480	0.480	0.480
membrane area	m^2^	0.23	0.23	0.23	0.23
channels number	-	42538	47087	54795	97083
Active area	m^2^	438	484	568	1003
O_2_ Volume Flux	NmL cm^−2^ min^−1^	2.598	2.352	2.001	1.180
Pressure loss feed	mbar	0.002	0.001	0.001	0.750
Pressure loss permeate	mbar	-	-	-	0.86
stack area	m^2^	11.6	11.6	11.6	11.5
stack volume	m^3^	0.37	0.37	0.37	0.37
stack number	-	38	42	49	87

**Table 4:** Comparison of cost assessment results for two considered oxy-fuel cases.

	B	C
Cost of plant *, k€	2378.7	2482.1
Yearly cost of fuel for the furnace, k€	1849.4	1839.4
Yearly cost of additional fuel, k€	-	448.3
Yearly cost of electricity, k€	410.2	−308.5
Discounted total cost, M€	17.54	15.76

Case B: oxy-fuel case with heat recover. Case C: oxy-fuel case with 3-end OTM system. * excepting the glass melting furnace and the after-treatment station.

## Nomenclature

***Symbols***	
a	Channel width/m
b	Channel height/m
C	Volumetric molar concentration/${\mathrm{mol}\;m}^{-3}$
$c_{k}$	Concentration of mobile charge carriers *k*/${\mathrm{mol}\;m}^{-3}$
$D_{ik}$	Binary diffusion coefficient of species *i* into *k*/$m^{2}s^{-1}$
$D_{h}$	Hydraulic diameter/m
$D_{Kn}$	Knudsen diffusion coefficient/$m^{2}s^{-1}$
$E_{act}^{f}$	Activation energy of forward reaction/${\mathrm{kJ}\;\mathrm{mol}}^{-1}$
$E_{act}^{r}$	Activation energy of reverse reaction/${\mathrm{kJ}\;\mathrm{mol}}^{-1}$
F	Faraday constant/$96,485.33{\;C\;\mathrm{mol}}^{-1}$
h	Specific molar enthalpy/${\mathrm{kJ}\;\mathrm{mol}}^{-1}$
$h_{m}$	Mass transfer coefficient/${m\;s}^{-1}$
$i_{k}$	Current density of charge carrier *k*/${A\;m}^{-2}$
$j_{i}$	Diffusive molar flux of species *i*/${\mathrm{mol}\;s}^{-1}{\;m}^{-2}$
$k_{f}$	Forward reaction rate constant/${\mathrm{kmol}\;m}^{-2}s^{-1}$
$k_{r}$	Reverse reaction rate constant/${\mathrm{kmol}\;m}^{-2}s^{-1}$
$k_{eq}$	Equilibrium constant of reaction/-
$M_{i}$	Molecular mass of species *i*/${\mathrm{kg}\;\mathrm{kmol}}^{-1}$
$N_{i}$	Absolute molar flux of species *i*/${\mathrm{mol}\;s}^{-1}{\;m}^{-2}$
p	Pressure/Pa
$\dot{Q}$	Thermal power/${\mathrm{kW}\;m}^{-3}$
R	Universal gas constant/$8314{\;J\;\mathrm{kmol}}^{-1}K^{-1}$
$r_{pore}$	Average pore radius/μm
r	Reaction rate/${\mathrm{mol}\;s}^{-1}{\;m}^{-3}$
Sh	Sherwood number/-
T	Temperature/K
t	Support thickness/mm
$x_{i}$	Molar fraction of species *i*/-
$v_{i}$	Velocity/${m\;s}^{-1}$
$z_{k}$	Number of charge carrier *k*/-
z	Transversal coordinate through supported membrane/-
***Greek symbols***	
$\delta_{m}$	Mass diffusion boundary layer/m
$\Delta H_{r}^{{}^{\circ}}$	Standard reaction enthalpy/${\mathrm{kJ}\;\mathrm{mol}}^{-1}$
ϵ	Average porosity/-
ϕ	Electrostatic potential/V
$\mu_{k}$	Chemical potential of charge carrier *k*/V
$\nu_{i}$	Molecule diffusion volume of species *i*/-
$\sigma_{k}$	Charge carrier *k* conductivity/${S\;m}^{-1}$
τ	Average tortuosity/-
***Acronyms***	
OTM	Oxygen Transport Membrane
MIEC	Mixed Ionic-Electronic Conductor
ORR	Oxygen Reduction Reaction
TPB	Triple Phase Boundary
DPB	Double Phase Boundary
SFM	Stagnant Film Model
DGM	Dusty Gas Model
SOFC	Solid Oxide Fuel Cells
VSA	Vacuum Swing Adsorption
PSA	Pressure Swing Adsorption
***Subscripts/Superscripts***	
w	Membrane wall
b	Gas bulk phase
mix	Mixture
′	Feed (reduction) side
″	Permeate (oxidation) side
eff	Effective property
Kn	Knudsen diffusion term

## Appendix A

This section reports for tables with details of the streams as numbered in Figure 11, Figure 12, Figure 13 and Figure 14, as well as details of some calculation assumptions for the fluid machinery.

**Table A1:** Mass flow rates, temperatures, pressures and compositions (mol.%) at the main stations in Figure 11 for a glass melting furnace with production of 90 ton day^−1^.

Stream	ṁ, kg h^−1^	T, °C	p, kPa	Ar	CO_2_	H_2_O	N_2_	O_2_
1	3750.0	15.0						
2	309.3	15.0		fuel (CH_4_: 89, C_2_H_6_: 7, CO_2_: 2, C_3_H_8_: 1.11; N_2_: 0.89)				
3	5559.8	15.0	101	0.92	0.03	1.03	77.28	20.73
4	5559.8	21.4	107	0.92	0.03	1.03	77.28	20.73
5	5559.8	935.2		0.92	0.03	1.03	77.28	20.73
6	5869.1	1313.7		0.84	8.86	17.54	70.76	2
7	8732.9	361.8		0.87	6.03	12.25	72.85	8
8	24828.1	46.6	93	0.84	2.12	11.20	70.86	14.98
9	24828.1	58.1	102	0.84	2.12	11.20	70.86	14.98

**Table A2:** Mass flow rates, temperatures, pressures and compositions (mol.%) at the main stations in Figure 12 for a glass melting furnace with production of 90 ton day^−1^.

Stream	ṁ, kg h^−1^	T, °C	p, kPa	Ar	CO_2_	H_2_O	N_2_	O_2_
1	3750.0							
2	235.7	450		fuel (CH_4_: 89, C_2_H_6_: 7, CO_2_: 2, C_3_H_8_: 1.11; N_2_: 0.89)				
3	983.2	15.0	101	4.00			4.00	92.00
4	983.2	21.5	107	4.00			4.00	92.00
5	983.2	550.0	104	4.00			4.00	92.00
6	1219.0	1313.7	101	2.76	32.04	60.17	3.03	2.00
7	1219.0	835.3		2.76	32.04	60.17	3.03	2.00
8	10804.0	50.9	93	1.08	3.71	13.88	64.03	17.31
9	10804.0	62.4	102	1.08	3.71	13.88	64.03	17.31
10	1306.4	15.0	101	0.92	0.03	1.03	77.28	20.73
11	1306.4	25.6	111	0.92	0.03	1.03	77.28	20.73
12	1306.4	750		0.92	0.03	1.03	77.28	20.73

**Table A3:** Mass flow rates, temperatures, pressures and compositions (mol.%) at the main stations in Figure 13 for a glass melting furnace with production of 90 ton/day.

Stream	ṁ, kg/h	T, °C	p, kPa	Ar	CO_2_	H_2_O	N_2_	O_2_
1	3750							
2	234.4	450.0		fuel (CH_4_: 89, C_2_H_6_: 7, CO_2_: 2, C_3_H_8_: 1.11; N_2_: 0.89)				
3	893.6	550.0	107					100.00
4	1128.0	1313.7	101		33.96	63.77	0.28	2.00
5	1128.0	800.0	101		33.96	63.77	0.28	2.00
6	1128.0	501.0	101		33.96	63.77	0.28	2.00
7	8690.4	15.0	101	0.92	0.03	1.03	77.28	20.73
8	57.1	15.0		fuel (CH_4_: 89, C_2_H_6_: 7, CO_2_: 2, C_3_H_8_: 1.11; N_2_: 0.89)				
9	8747.6	950.0	400	0.91	1.16	3.14	76.45	18.34
10	7853.8	950.0	400	1.00	1.28	3.46	84.17	10.10
11	7853.8	648.0	103	1.00	1.28	3.46	84.17	10.10
12	893.6	40.0	24					100.00
13	5583.6	58.3	93	0.68	7.06	19.76	56.85	15.65
14	5583.6	69.8	102	0.68	7.06	19.76	56.85	15.65

**Table A4:** Mass flow rates, temperatures, pressures and compositions (mol.%) at the main stations in Figure 14 for a glass melting furnace with production of 90 ton day^−1^.

Stream	ṁ, kg h^−1^	T, °C	p, kPa	Ar	CO_2_	H_2_O	N_2_	O_2_
1	3750.0							
2	260.8	15.0		fuel (CH_4_: 89, C_2_H_6_: 7, CO_2_: 2, C_3_H_8_: 1.11; N_2_: 0.89)				
3	2280.8	950.0		0.04	40.64	5.46	3.86	50.00
4	2541.5	1313.7		0.03	52.78	41.91	3.28	2.00
5	2541.5	900.0		0.03	52.78	41.91	3.28	2.00
6	2541.5	250.0		0.03	52.78	41.91	3.28	2.00
7	2019.1	40.0	93	0.05	83.65	7.93	5.2	3.17
8	2019.1	49.0	102	0.05	83.65	7.93	5.2	3.17
9	32.7	15.0	101	0.92	0.03	1.03	77.28	20.73
10	1260.3	61.7	111	0.09	80.62	7.68	7.81	3.81
11	1260.3	700.0	109	0.09	80.62	7.68	7.81	3.81
12	9.4	15.0		fuel (CH_4_: 89, C_2_H_6_: 7, CO_2_: 2, C_3_H_8_: 1.11; N_2_: 0.89)				
13	1269.7	950.0	107	0.08	81.01	10.87	7.69	0.34
14	9862.2	192.1	434	0.92	0.03	1.03	77.28	20.73
15	9862.2	330.8	425	0.92	0.03	1.03	77.28	20.73
16	9862.2	550.0	416	0.92	0.03	1.03	77.28	20.73
17	9862.2	700.0	408	0.92	0.03	1.03	77.28	20.73
18	66.4	15.0		fuel (CH_4_: 89, C_2_H_6_: 7, CO_2_: 2, C_3_H_8_: 1.11; N_2_: 0.89)				
19	9928.8	950.0	400	0.91	1.19	3.19	76.43	18.28
20	8917.6	950.0	400	1.00	1.31	3.52	84.12	10.06
21	8917.6	648.1	103	1.00	1.31	3.52	84.12	10.06
22	8917.6	415.5	101	1.00	1.31	3.52	84.12	10.06

Two blowers are present in the layout of the reference case A. Auxiliary energy consumptions are calculated based on:

- a pressure ratio of 1.06 for the blower C1 (air to the burners);
- a pressure ratio of 1.1 for the blower C2 (extraction of the gas from the after-treatment station).

Isentropic and mechanical-electrical efficiency values equal to 0.75 and 0.9, respectively, are assumed. Thus, the calculated energy consumptions result in 11.2 kW for the blower C1 and 93.9 kW for the blower C2.

Three main auxiliaries are present in Case B. According to:

- a pressure ratio of 1.06 for the blower C1 (oxygen to pre-heater then to burners);
- a pressure ratio of 1.1 for the blower C2 (extraction of the gas from the after-treatment station);
- a pressure ratio of 1.1 for the blower C3 (delivering the hot carrier through the regenerative heat exchanger for oxygen and fuel pre-heating);

Electric energy consumptions of 1.8 kW, 41.3 kW and 4.3 kW are calculated respectively for the three blowers. The auxiliary consumptions total 47.3 kW. In addition, the energy demand related to the oxygen production must be included. Based on the calculations, slightly less than 700 Nm^3^ h^−1^ are required, so the oxygen can be produced by pressure or vacuum swing adsorption technology instead of a small cryogenic distillation process. Assuming an electric consumption of 0.4 kWh per Nm^3^ of oxygen, along with an electric conversion efficiency of 50%, the related electric consumption will result equal to 275.2 kW.

As regards the advanced oxy-fuel case with OTM technology in Figure 13, four main auxiliaries are present. The following assumptions have been made:

- pressure ratios of 4.43 and 1.1 for the vacuum pump C1 and the blower C2, respectively, with the previously considered efficiency values;
- a pressure ratio of 4.2 with an isentropic efficiency of 83% for the compressor C3;
- a pressure ratio of 3.88 with an isentropic efficiency of 87% for the turbine T1;
- a mechanical-electrical efficiency of 0.9 for the micro-gas turbine including the compressor C3 and the turbine T1.

The electric power consumptions have resulted equal to 55.2 kW and 21.9 kW for the vacuum pump C1 and the blower C2, respectively, whereas a net power of 319.6 kW has been calculated for the micro-gas turbine system.

Ultimately, always four machineries are present in the schematic of the advanced oxy-fuel case with OTM technology in Figure 14. The following assumptions have been made:

- pressure ratios of 1.1 for both the blowers C1 and C2, with the previously considered efficiency values;
- a pressure ratio of 4.28 (differently from the case in Figure 13, one more passage through heat exchanger is considered here) with an isentropic efficiency of 83% for the compressor C3;
- a pressure ratio of 3.88 with an isentropic efficiency of 87% for the turbine T1;
- a mechanical-electrical efficiency of 0.9 for the micro-gas turbine including the compressor C3 and the turbine T1.

The electric power consumptions have resulted equal to 3.3 kW and 5.1 kW for the blowers C1 and C2, respectively, whereas a net power of 374.2 kW has been calculated for the micro-gas turbine system.
